# Posttransplant Intrarenal Lymphangiectasia

**DOI:** 10.1155/2020/8824833

**Published:** 2020-07-21

**Authors:** Ali Kord, Enrico Benedetti, James T. Bui

**Affiliations:** ^1^Division of Interventional Radiology, Department of Radiology, University of Illinois, College of Medicine, Chicago, IL, USA; ^2^Department of General Surgery, University of Illinois, College of Medicine, Chicago, IL, USA

## Abstract

Renal lymphangiectasia is an extremely rare benign condition in the setting of transplanted kidneys. We describe a 50-year-old female with a past medical history of lupus nephritis and renal transplants who presented with right lower quadrant pain and was found to have intrarenal lymphangiectasia on imaging and laboratory tests. The patient was treated with percutaneous drainage initially and then wide peritoneal fenestration and omentoplasty. An extremely rare adult case with intrarenal lymphangiectasia thirteen months after kidney transplant was described in this study. Imaging, particularly computed tomography (CT) and magnetic resonance imaging (MRI), plays a key role in the diagnosis of renal lymphangiectasia.

## 1. Introduction

Renal lymphangiectasia is a rare benign condition [1], even more so in the setting of renal transplantation [2]. The condition can be intrarenal or extrarenal with dilated lymphatics seen in the peripelvic versus perinephric regions, respectively [3]. There have been only two reported renal transplant cases with extrarenal lymphangiectasia in which both donors were pediatric and one recipient was an adult [2]. In this study, we present an extremely rare adult case of intrarenal lymphangiectasia thirteen months after kidney transplant.

## 2. Case

**Figure 1:**
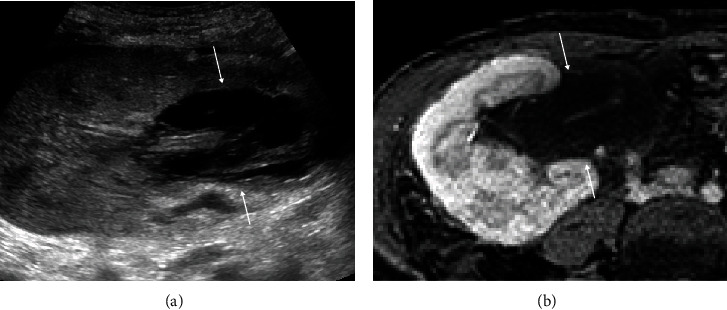
Right lower quadrant transplanted kidney. Ultrasound shows a complex, septated collection in the renal pelvis mimicking hydronephrosis (arrows, (a)) which was confirmed on the corresponding postcontrast fat-saturated axial MRI (arrows, (b)).

**Figure 2:**
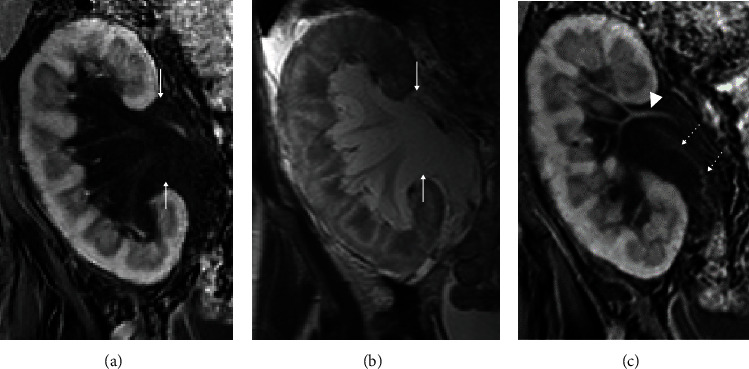
Coronal MRI of transplanted kidney. Postcontrast fat-saturated (a, c) and T2-weighted (b) show a nonenhancing peripelvic multiseptated fluid collection centered in the renal hilum which is hypointense on T1-weighted images and hyperintense on T2-weighted images (arrows, (a, b)). The fluid collection is encasing the hilar vasculature (arrowhead, (c)) and insinuating parallel to the ureter (dotted arrow, (c)) but is separate from the collecting system. There is no vascular displacement or hydronephrosis.

A 50-year-old female with past medical history of lupus nephritis and three renal transplants presented with right lower quadrant pain thirteen months after the last kidney transplant. On physical examination, the patient was mildly tender on the right lower quadrant. The laboratory tests showed increased creatinine at 264 *μ*mol/L (2.99 mg/dL). The patient underwent ultrasound which demonstrated increased cortical echogenicity of the transplant kidney, suggestive of medical renal disease without hydronephrosis. A multiseptated thin-walled fluid collection was present in the hilum of the transplanted kidney which was separate from the transplant collecting system (Figure 1(a)). Magnetic resonance imaging (MRI) of the transplant kidney was performed and revealed a nonenhancing peripelvic multiseptated fluid intensity collection centered in the renal hilum, encasing the hilar vasculature and insinuating between but separate from the collecting system without vascular displacement or hydronephrosis (Figures 1(b) and 2).

The differential diagnoses for delayed perirenal transplant fluid collections include seroma, urinoma, hematoma, abscess, or of lymphatic source. After initial identification with ultrasound, MRI was performed to better assess the extent of the fluid and its relationship to the collecting system. Rather than displace or cause mass effect, the fluid insinuated along and between the collecting system, vessels, and renal hilum. The imaging and clinical features in our case were not typical for hematoma or abscess. It may be difficult to differentiate urinoma or seroma from lymphangiectasia based on the imaging feature only, and fluid sampling is usually required. Diagnostic and therapeutic percutaneous catheter drainage was performed yielding clear fluid with negative culture. The cell count showed elevated lymphocytes (lymphocytes: 80, neutrophils: 1), and the fluid analysis did not show increased creatinine compared to the concurrent serum creatinine (fluid: 256 *μ*mol/L, serum: 264 *μ*mol/L) ruling out urinoma as a possible cause. These findings were most compatible with intrarenal lymphangiectasia. The patient's symptoms improved after drain placement, but drainage output was greater than one liter per day. After persistent high-volume drainage output for 8 weeks, the patient underwent wide peritoneal fenestration and omentoplasty. The patient was followed regularly at the outpatient clinic four months after the surgery with resolved symptoms.

## 3. Discussion

Renal lymphangiectasia is a rare benign disease which is believed to be caused by miscommunication between the renal and retroperitoneal lymphatics. This may result in dilatation and ectasia of the lymphatic vessels and formation of parenchymal or perinephric collections [1, 4]. Patients with renal lymphangiectasia may be asymptomatic or may present with abdominal distension, flank pain, abdominal lump, hypertension, hematuria, proteinuria, or renal vein thrombosis [1]. The extrinsic compression may result in ureteric obstruction or altered kidney function and can be confused for hydronephrosis or simple perinephric fluid collection posttransplantation [5]. The patient in our study presented with right lower quadrant pain and worsening kidney function thirteen months after kidney transplant.

The clinical presentation of the renal lymphangiectasia is nonspecific, and imaging and laboratory tests play an important role in diagnosis. Ultrasound usually shows an enlarged kidney with an anechoic multiseptated thin-walled fluid collection with possible increased renal cortical echogenicity [1]. Cross-sectional imaging including computed tomography (CT) and MRI may demonstrate cystic dilatation of the renal sinuses, not connected to the collecting system [6–8]. The imaging in the previous two cases with transplant renal lymphangiectasia including ultrasound and CT showed subcapsular and perinephric fluid collections compatible with extrarenal lymphangiectasia [2]. The ultrasound and MRI in our case, in contrast, demonstrated nonenhancing peripelvic multiseptated fluid collections centered in the renal hilum, encasing the hilar vasculature and insinuating between but separate from the collecting system without vascular displacement or hydronephrosis. On histological examination, renal lymphangiectasia is usually positive for lymphatic endothelial immunomarkers, including D2-40, and is characterized by cortical dilated endothelial-lined spaces, without glomerular or tubular abnormalities [9]. Similar to our patient, the fluid analysis may show elevated lymphocytes and triglyceride [10].

Asymptomatic patients with renal lymphangiectasia can be followed to detect early kidney dysfunction. Symptomatic patients can be treated with aspiration and possibly surgical peritoneal fenestration, given high recurrent rate and relative contraindication of sclerosing therapy due to potential stenosis of the collecting system [11]. The mTOR inhibitors have been reported to be effective in the treatment of intestinal lymphangiectasia [12] and may be beneficial for intrarenal lymphangiectasia [2], although not used in our patient. Nephrectomy is very rarely performed and mainly reserved for recurring complicated collections and uncontrolled intraoperative bleeding [1, 13]. The two previously reported cases with transplant renal lymphangiectasia were initially treated with conservative medical treatment, therapeutic paracentesis, and ablation and sealing of the lymphatic leak but finally required multiple laparoscopic and open operative procedures and underwent allograft nephrectomy due to relapsing ascites and significant impact on the patient's quality of life [2]. Our patient underwent wide peritoneal fenestration and omentoplasty due to persistent high output from the percutaneous drain. In contrast to the previous two transplant cases with extrarenal lymphangiectasia, our patient did not present with ascites and showed resolved symptoms four months after the surgery.

In summary, an extremely rare adult case of intrarenal lymphangiectasia thirteen months after kidney transplant was described. Imaging, particularly CT and MRI, play a key role in diagnosis of renal lymphangiectasia and preprocedural planning.

## Data Availability

The clinical data used to support the findings of this study are restricted by the University of Illinois Hospital in order to protect Protected Health Information. Data are available from the corresponding author (JB: jtbui@uic.edu) for researchers who meet the criteria for access to confidential data.
